# Seeking unique and common biological themes in multiple gene lists or datasets: pathway pattern extraction pipeline for pathway-level comparative analysis

**DOI:** 10.1186/1471-2105-10-200

**Published:** 2009-06-29

**Authors:** Ming Yi, Uma Mudunuri, Anney Che, Robert M Stephens

**Affiliations:** 1 Advanced Biomedical Computing Center, Advanced Technology Program, SAIC-Frederick Inc, NCI-Frederick, Frederick, MD 21702, USA

## Abstract

**Background:**

One of the challenges in the analysis of microarray data is to integrate and compare the selected (e.g., differential) gene lists from multiple experiments for common or unique underlying biological themes. A common way to approach this problem is to extract common genes from these gene lists and then subject these genes to enrichment analysis to reveal the underlying biology. However, the capacity of this approach is largely restricted by the limited number of common genes shared by datasets from multiple experiments, which could be caused by the complexity of the biological system itself.

**Results:**

We now introduce a new Pathway Pattern Extraction Pipeline (PPEP), which extends the existing WPS application by providing a new pathway-level comparative analysis scheme. To facilitate comparing and correlating results from different studies and sources, PPEP contains new interfaces that allow evaluation of the pathway-level enrichment patterns across multiple gene lists. As an exploratory tool, this analysis pipeline may help reveal the underlying biological themes at both the pathway and gene levels. The analysis scheme provided by PPEP begins with multiple gene lists, which may be derived from different studies in terms of the biological contexts, applied technologies, or methodologies. These lists are then subjected to pathway-level comparative analysis for extraction of pathway-level patterns. This analysis pipeline helps to explore the commonality or uniqueness of these lists at the level of pathways or biological processes from different but relevant biological systems using a combination of statistical enrichment measurements, pathway-level pattern extraction, and graphical display of the relationships of genes and their associated pathways as Gene-Term Association Networks (GTANs) within the WPS platform. As a proof of concept, we have used the new method to analyze many datasets from our collaborators as well as some public microarray datasets.

**Conclusion:**

This tool provides a new pathway-level analysis scheme for integrative and comparative analysis of data derived from different but relevant systems. The tool is freely available as a Pathway Pattern Extraction Pipeline implemented in our existing software package WPS, which can be obtained at

## Background

Microarray and other high throughput (HTP) technologies have exponentially increased in popularity in recent years and consequently have generated tremendous amounts of data. This data provides great opportunities for systems-level understanding of the underlying biological themes of complex experiments. As a result, a wide range of software tools that process and analyze the data using different approaches and algorithms have been developed including clustering methods (e.g., hierarchical [1]; K-means [2], SOM [3]) methods, pattern extraction method [4], identifying differential gene lists from two or more classes contrasts (e.g., Significance Analysis of Microarray [5], LPE [6], and analysis of variance (ANOVA) related methods [7,8]). In order to place these patterned or differential genes into their biological contexts, they can be mapped into pathways or networks for further analysis of biological associations and relationships among them as well as other documented relevant genes curated from the literature [4,9-11]. Alternatively, functional group or gene set overrepresentation analysis (ORA) methods [12-14] or gene set-based enrichment analysis (e.g., GSEA [15,16]) can be used to identify the significantly affected pathways or gene sets that are enriched or over-represented within a list of patterned or differential genes.

The enormous increase in availability of data from studies using similar biological systems, independent samplings, and/or technical platforms (e.g., microarray, proteomics) allows an integrative and comparative analysis to be performed. This provides for a deeper understanding of the underlying biology and consolidation of initial observations made from individual studies. Furthermore, the systems-oriented approach allows for additional insights from combined datasets. For example, diseases such as prostate cancer have been studied by many different groups. These data from different platforms and independent samplings provide the opportunity not only to assess the consensus of these studies and the variation levels of the patient population, but also to perform integrative analysis for signatures at both gene and pathway level.

A conventional way to integrate and compare multiple experiments derived from independent research groups or even different technologies for common or unique underlying biological themes is to derive common genes amongst them before subjecting them to enrichment analysis to reveal the underlying biology. However, such an approach often encounters limitations caused by the diversity of technologies and the complexity of the biological system itself.

Many of the available software tools primarily retrieve expression patterns at the individual gene level and generate a list of genes that are differentially expressed or have certain expression patterns across samples. Even the software tools that employ ORA or GSEA methods [14-16], usually consider only one or very limited number of gene lists at a time. Although a great deal of attention was placed on gene-level expression patterns initially, there is an urgent need for capturing the pathway-level patterns that may represent the common or unique biological themes, which are embedded in multiple genes lists or multiple datasets from different, but related studies.

It has become more and more evident that gene level signatures or classifiers that can consistently characterize different tumor types are relatively hard to validate across different studies due to the complexity of the underlying biology (e.g., large genetic variations within the phenotypical population), experimental variation, and even the choice of data processing (e.g., normalization, transformation) and analysis methods/algorithms. This observation likely results from the fact that many complex diseases including cancers, heart disease and hypertension have been shown to be caused by mutations in multiple genes in the same or related pathways [17-20]. While biologically relevant genes may consistently behave in correlation with an associated phenotype across a population, it is more likely that common pathways can be impacted through distinct gene events that are not reflected at the individual gene level. This seems particularly relevant considering the stochastic nature of many epigenetic events that lead to disease states. For example, evidence derived from the studies on multiple prostate datasets of different research groups has shown that many pathway/gene set enrichment analysis or group testing methods identified the same or related pathways/gene sets that were either experimentally validated or believed to be more likely involved in the pathogenesis of prostate cancer. Furthermore, these pathways/gene sets appear to be more consistent and reproducible than simple gene signatures between these datasets [21]. These, and similar observations have fueled efforts to evolve from gene-level signatures into pathway-level signatures. Similarly, elevation of gene-based classification methods into pathway-based classification methods is now being actively pursued in the field. The gene-by-gene approach or gene-level paradigm failed to put single genes in an overall functional context, and consequently ignored other relevant genes that were known to have biological relevance or showed similar expression profiles or correlation with the phenotypes under study. Now that independent studies of the same or similar subjects produced gene lists with very low levels of overlap [21], it makes more sense to compare these gene lists at the pathway-level for common and unique biological significance.

Using this rationale as our motivation, we developed a high-throughput Pathway Pattern Extraction Pipeline (PPEP) within the existing pathway analysis tool WPS [4], to specifically address the need for pathway-level comparative analysis using pathway-level enrichment patterns. The method can explore the biological commonality or uniqueness at the level of a pathway or biological process from different but relevant biological systems. With the help of PPEP, we can intuitively visualize the patterns of pathway-enrichment across the multiple gene lists or datasets The method also reveals the relationships and data behavior of the involved genes from the gene lists or datasets that are associated with these pathways.

As a proof of concept of this exploratory tool, we have used PPEP to analyze both public microarray datasets and datasets from our collaborators. In summary, we found that this new analysis pipeline dramatically enhanced the ability of research investigators to uncover the underlying biological themes that are shared or unique among different but related systems represented by these genes lists or datasets through pathway-level comparative analysis.

## Implementation

### Pathway pattern extraction pipeline dissects pathway-level enrichment patterns for biological themes

The first version of WholePathwayScope or WPS provides a platform for pattern extraction at the gene-level and also allows generation of gene-term association networks (GTANs) [4]. In this newly developed high-throughput pathway pattern extraction pipeline (PPEP), we extended that capacity into a pathway-level pattern extraction method that retrieves the patterned pathways based on enrichment levels of pathways across gene lists or datasets. From the patterned pathways, the associated genes can be retrieved and displayed in the context of GTANs [4]. The objective of such a method is to combine pathway-level enrichment analysis, heatmap/clustering analysis, and pattern extraction to look for pathway-level enrichment patterns as underlying biological themes.

PPEP is implemented within the existing WPS software platform and runs in the WPS environment. It utilizes an enhanced WPS internal database with more species coverage (now including yeast, and malaria) and more functional categories including GSEA gene sets, predicted transcription factor target genes, and miRNA target genes for human and mouse. The goal of PPEP is to perform pathway-level comparative analysis of underlying biology represented by multiple lists of signature genes, differential genes, or patterned genes derived from different studies, different groups, and/or even different technologies and platforms. In our current release of WPS, PPEP works side by side with our newly developed pathway-ranking method-SLEPR [22] forming the main pathway-level analysis core utilities in WPS. Although our current version of WPS is a Window-specific application, we are currently planning to port this application to Java so that the operating system restriction will be removed.

**Figure 1:**
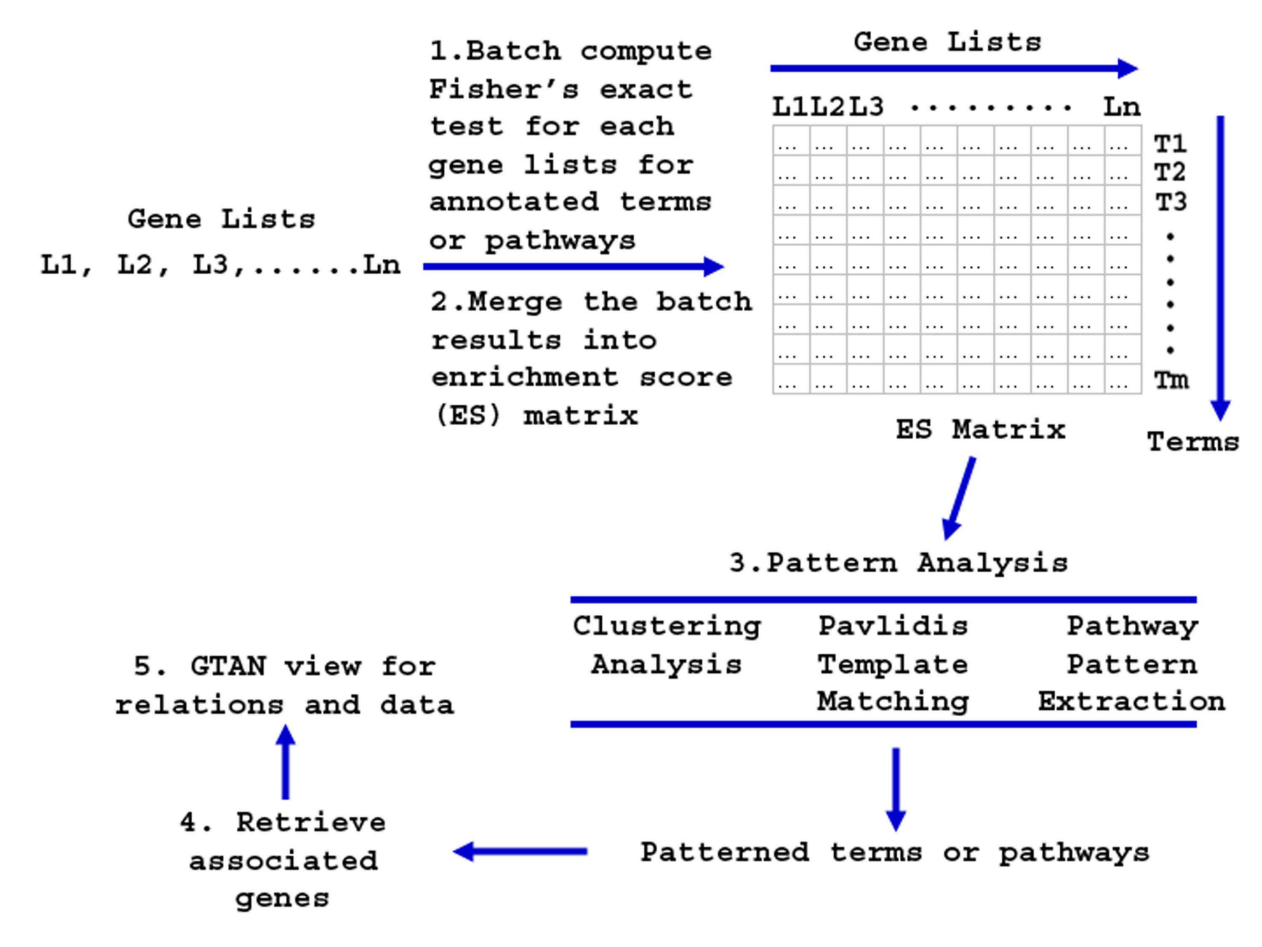
**Schematic work flow of Pathway pattern extraction Pipeline (PPEP) in WPS**. The input of the analysis pipeline can be a set of gene lists derived from the same or even different study in terms of biological contexts, applied technologies and methodologies. Examples of the lists are differential gene lists from class contrasts obtained from microarray data analysis, proteomic profiling, or genome-wide post-transcriptional modification scan. Then the first step is to batch compute the Fisher's exact test to evaluate the enrichment levels for each gene lists for annotated terms or pathways in WPS database (e.g., GOBP, Biocarta pathway). The Fisher's exact test for enrichment levels was described previously [4]. Step 2 is to collect the batch computation results and merging them into an enrichment score (ES) matrix. Then at step 3, the ES matrix can be subjected to pattern analysis using different methods under user's choice: clustering analysis, Pavlidis template matching, and pathway pattern extraction. Clustering analysis and Pavlidis template matching can be done with external tools such as TM4 package from TIGR. The pathway pattern extraction is a newly designed feature in PPEP specifically for pattern extraction using ES score matrix. Then the next step or step 4, obtained terms or pathways after pattern analysis, usually as in a matrix or as a list, can be used to retrieve the associated genes using the interface of the analysis pipeline in WPS. Then at the final step, the data and association relation of the terms and genes can be viewed under GTAN display in WPS using WPS visualization capacity.

As illustrated in Figure 1, PPEP consists of several functional interfaces that allow a designated analysis procedure to be executed in a step-by-step fashion. The modular design through multiple interfaces allows the user to assess results at each stage and then proceed as desired.

**Figure 2:**
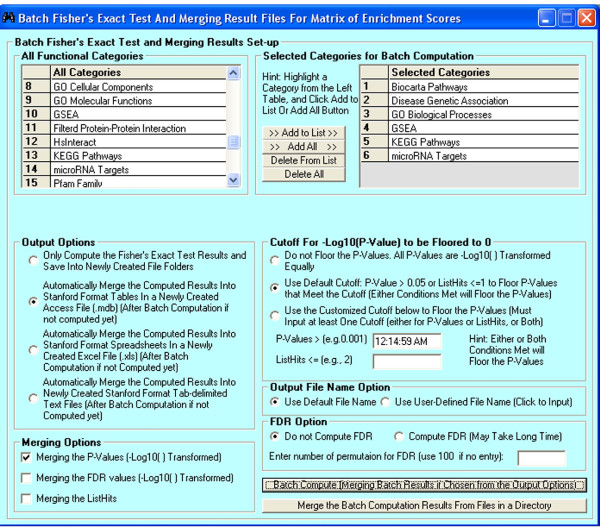
**The interface of the PPEP for batch computation of Fisher's exact test of multiple gene lists and merging the result files for matrix of enrichment scores**. From each species-specific database, multiple functional categories can be selected for batch computation. Users have options merging p-values for enrichment scores, merging ListHits (how many genes from the lists hit the term), or merging -log10 transformed FDR values into a matrix. As default, Fisher's exact test p-values can be floored by criteria of p > 0.05 and ListHits <= 1 to make the corresponding enrichment scores as 0. However, users have options to either not floor the enrichment scores, or set up their own customized way to do the flooring. Other options are also provided: e.g., computing FDRs or not, or number of iterations for FDR computations.

**Figure 3:**
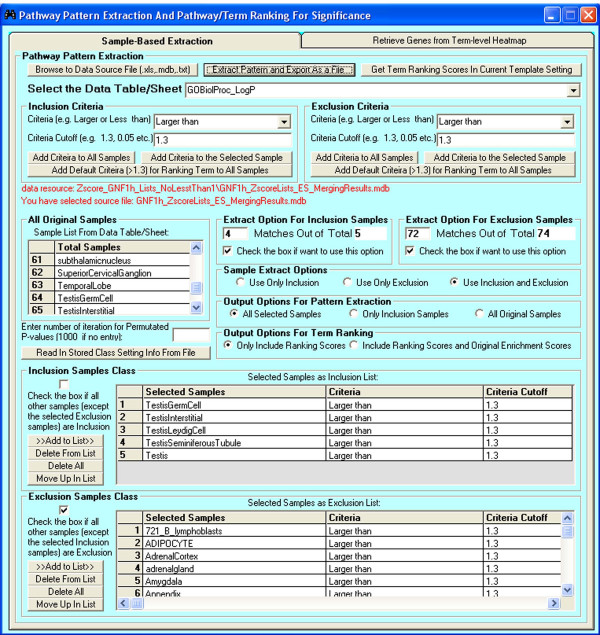
**The interface for pathway pattern extraction of the PPEP**. User can browse to the data sources (e.g., a matrix of enrichment scores) in multiple file formats (text file, Microsoft Excel file and Access file), select Inclusion samples and Exclusion samples from sample list, set criteria for both Inclusion and Exclusion samples. There are also Extract Options for both Inclusion and Exclusion samples so that a term with a portion of samples meets the criteria can be selected.

**Figure 4:**
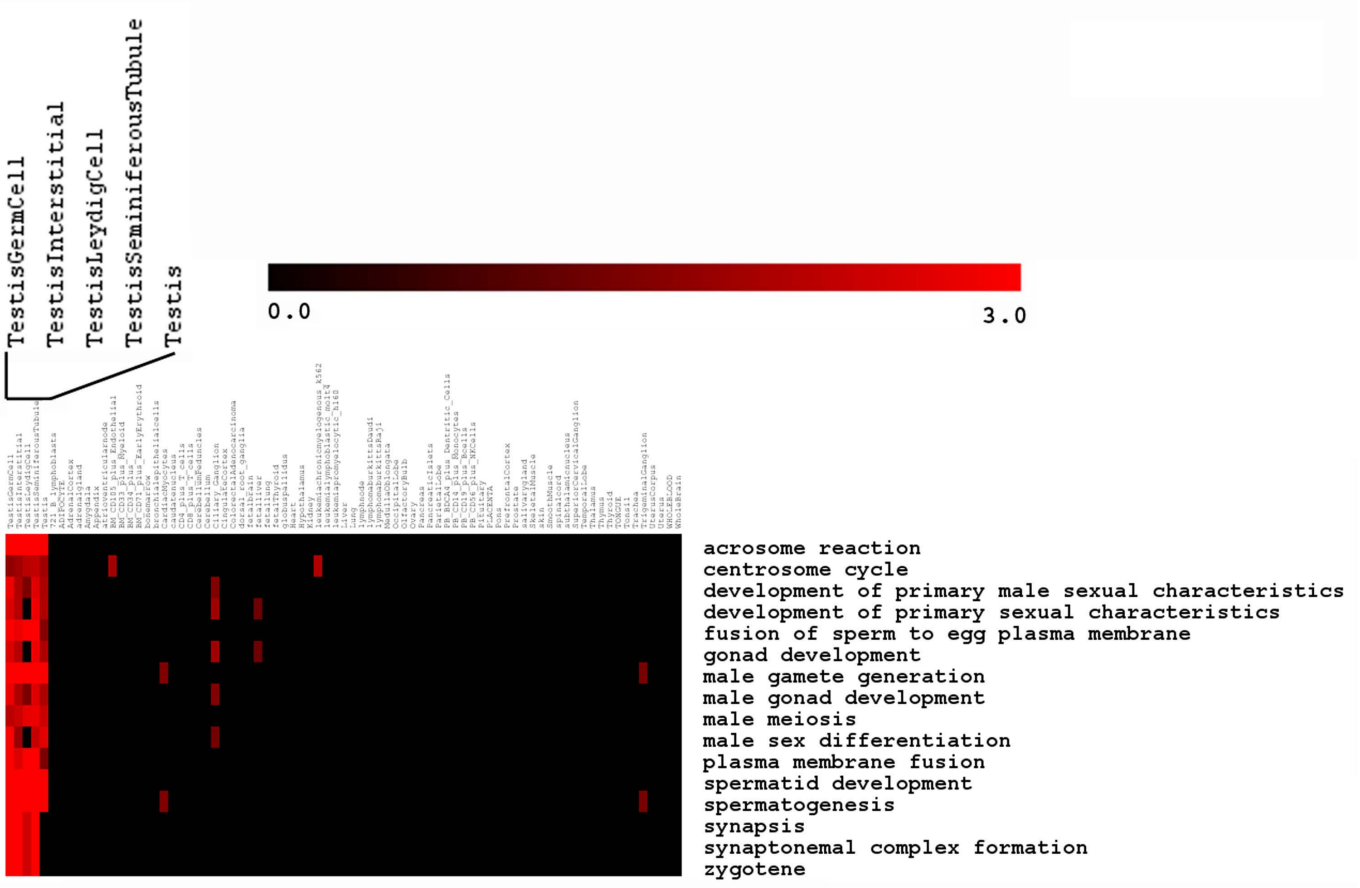
**An example of pathway pattern extraction results in a heatmap**. The data of patterned GO biological process terms for GNF dataset [43] was obtained with pathway pattern extraction, whose extraction template was set as shown in Figure 3: look for patterned terms that are enriched in at least 4 of 5 Inclusion samples (testis-related tissues: testis germ cells, testis interstitial, testis leydig cells, testis seminiferous tubule, testis), but not enriched in at least 72 of 74 Exclusion samples (the rest of tissues in GNF dataset).

**Figure 5:**
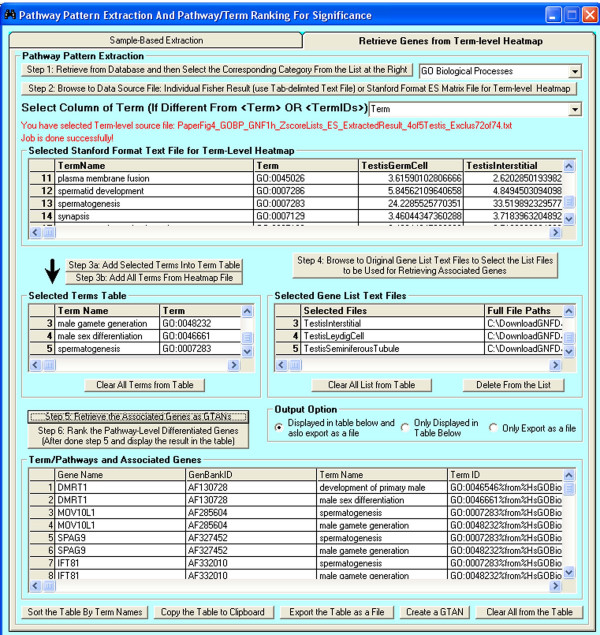
**The interface of the PPEP for retrieval of associated genes with selected terms from pattern extraction results or other resources**. Users first need to select the functional category for the intended terms (e.g., GO Biological Processes). Then the data source (i.e. a Stanford format file with the matrix of terms [4]) can be selected, from which intended terms can be selected. The gene list files that are originally used for batch computation of enrichment results can be selected into the <Selected Gene List Text Files> table. The retrieved gene-term relations are presented in a binary format (gene-term pairs), which can be either displayed in the table at the bottom of the interface, or saved as a file for future use. The retrieved pairwise gene-term relations displayed in the table can be immediately used to create a GTAN (gene-term association network: [4]) and visualize the data of the interested gene lists on the context of GTANs.

**Figure 6:**
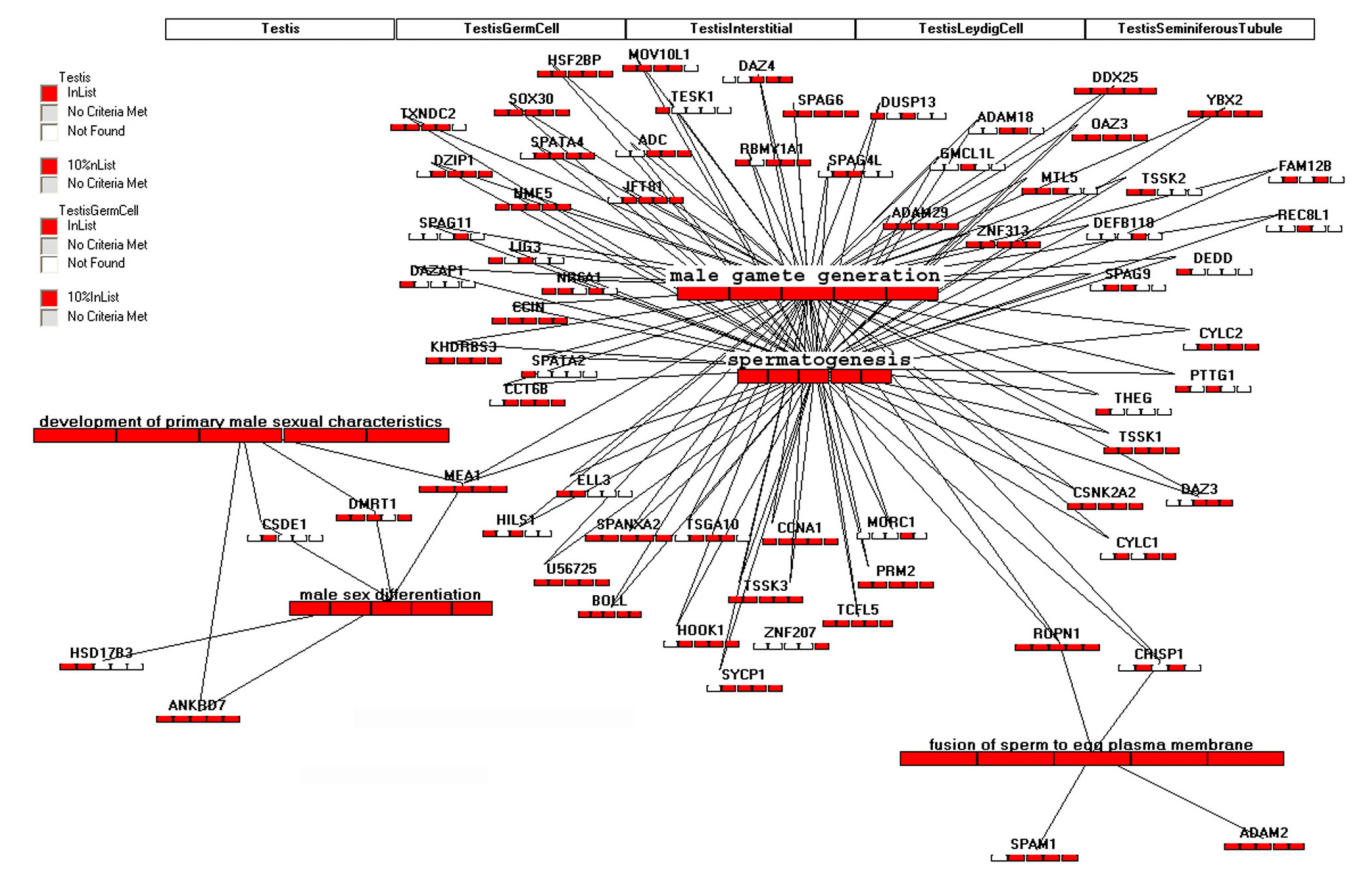
**An example of GTAN view of the retrieved associated genes with the patterned terms derived from pathway pattern extraction**. The associated genes of some selected GO biological process terms extracted from GNF dataset by pathway pattern extraction (Figure 4) was retrieved with the gene retrieval interface shown in Figure 5. The red color for genes shows that these genes are in the original gene lists of corresponding samples of testis-related tissues. The red color for pathways shows that there are more than 10% of associated genes for the corresponding terms, which are in the original gene lists of corresponding samples of testis-related tissues.

The generic step-by-step procedure for PPEP is described as follows (Figure 1). First, multiple gene lists from different sources are subjected to batch computation for enrichment analysis for selected functional categories (Figure 2). The enrichment analysis is performed using Fisher's exact test, as described previously [4]. The result for each list is written into an individual file. Next, the p-values of each list for each term in these result files can be transformed and merged together as a matrix of enrichment scores (ESs) in a single Stanford format file [4], in which each row is a term and each column is a gene list. Alternatively, FDR and ListHits can be merged together in a similar way. For step 3, the matrix of ESs can be subjected to clustering analysis using many existing common clustering analysis tools [23,24], PTM-based pattern extraction using TM4 from TIGR [23], or pathway pattern extraction within PPEP (Figure 3 and 4). Then at step 4, the associated genes can be retrieved from the patterned pathways (enrichment-level based) (Figure 5). Finally, the retrieved genes can be displayed in the context of GTANs, as described previously [4] (Figure 6). The more detailed description on the features and implementation of PPEP is included into the Additional file 1 (Additional file 2, 3, and 4).

### Data analysis using PPEP

The PPEP in WPS provides an opportunity to study the underlying common or unique biological themes among multiple gene lists derived from related but different systems or class comparisons. It can also be used to assess different datasets with the same or even different microarray platforms or HTP datasets using other technologies that measure different aspects of the biological systems. For example, one could ask what can be found in common between different studies of the same disease, since it has been found in many cases that only a few common genes are found for similar studies or for the same study but with different analysis methods [21,25]. However, the levels of consistency could be higher and more obvious when evaluated at the pathway level. The result section of this manuscript illustrates case studies with the PPEP using real data examples to describe some analysis schemes to seek common or unique biological themes embedded in different studies.

## Results

The major features and functionalities of the newly developed pathway pattern extraction pipeline (PPEP) in WPS are illustrated using example data in the following section. Although only microarray data is utilized here as examples, any source of HTP data would be equally suitable for the analysis with PPEP. More examples are also provided as supplementary files.

### Seeking common biological themes from different studies on the same subjects

One of the major advantages of PPEP is the ability to compare the biological themes for multiple gene lists derived from different studies at the pathway-level. To provide examples of such an analysis scheme, we chose to use the prostate cancer datasets derived from different research groups and collected in the Oncomine database [26]. This allows different studies on the same subjects to be compared at both the gene and pathway levels. The reason we chose prostate cancer is that this type of cancer has been well studied and there are many microarray datasets available on different platforms and from different research groups. Our analysis goal is to compare the differential genes derived from the different studies and to seek the common biological themes as the consensus (e.g., pathways, GO terms, gene sets) that are embedded in these differential gene lists.

From the Oncomine database, we downloaded a total of 6 microarray datasets that all specifically studied prostate cancer [27-32] but with different microarray platforms (e.g., Affymetrix, cDNA array, customized array etc.). The Oncomine database [26] has done gene-level analysis on each of these datasets and reported the differential gene lists (at Q-value < 0.05) between prostate tumor and normal or benign tissues. We can immediately subject these lists to analysis with PPEP. Since the same type of analysis was applied to all of the datasets in the Oncomine database, it is reasonable to assume that the statistical criteria to generate the differential genes are consistent for all of the datasets such that the derived differential gene lists can be compared at pathway-level directly.

Conventionally, to identify the common elements between datasets from different studies, the shared differential genes can be obtained, from which the biological themes can be deduced using enrichment analysis methods [12,13]. It has also been reported that analysis at the pathway level can improve the comparability of different datasets [21]. Our PPEP method is intended to improve the comparability of different datasets by simply extracting pathway enrichment patterns.

To compare the conventional gene-level analysis method with our pathway-level pattern extraction method implemented in the PPEP, we first used WPS gene pattern extraction feature to retrieve 52 genes, which are differentially expressed between prostate tumor and normal tissues and are commonly shared among all of the 6 datasets (see Additional file 5). Then we subjected the list of these 52 genes (we called it 52-gene common gene list below) to enrichment analysis in WPS from both GO (Gene Ontology) Biological Processes (GOBP) and GSEA annotation. Conventionally, the enriched terms from the 52-gene common gene list would be treated as the common biological themes among the 6 independent datasets. To compare results with the conventional method, we directly used our PPEP to do pathway pattern extraction using the differential gene lists from all of the 6 datasets for GOBP and GSEA annotations. For GSEA annotations, our pathway pattern extraction method obtained 331 terms that are significantly enriched (P < 0.05 in each dataset) consistently in differential gene lists of all 6 datasets (see Additional file 6). When the 52-gene common gene list was used in the conventional enrichment analysis for GSEA annotations, only 125 terms are significantly enriched (see Additional file 7). Furthermore, among the commonly enriched 331 terms that we obtained by pathway pattern extraction, there are 33 terms directly involved in cancer-related processes, which are shown in the heatmap (see Additional file 8). Among these 33 cancer-related terms, only 7 are significantly enriched in the 52-gene common gene list (see Additional file 9). Similarly, there are only 22 GO terms that are significantly enriched in the 52-gene common gene list, while there are 44 GOBP terms derived from pathway pattern extraction that are enriched consistently in the differential gene lists (see Additional file 10 and 11). After taking a close look at these GOBP terms, although there were many apoptosis-related GOBP terms repeatly appearing in the patterned term lists obtained from the PPEP (see Additional file 12), there were no apoptosis or cell death related terms enriched in the 52-gene common gene list (see Additional file 11). Perturbation of apoptosis-related processes is well known to play critical role in carcinogenesis. These observations suggested that our pathway pattern extraction method provided by the PPEP was able to uncover many of the significant terms, which were missed by the conventional gene-level based enrichment approach. The pathway pattern extraction method may be much more powerful than conventional gene-level based approach at revealing the embedded biological themes. Importantly, by identifying additional terms, the PPEP method provides the researcher with more information to consider with their own biological domain expertise.

In order to evaluate how useful pathway pattern extraction would be from a generic perspective, we also used PPEP to look for higher level enrichment patterns for transcription factor targets, miRNAs targets and KEGG pathways. We extracted 14 potential transcription factors with binding sites enriched consistently in each of the differential gene lists from all 6 datasets (see Additional file 13). Among these transcription factors, AP-2 [33], SP1 [34], and NF-kB [35] are either well known or implicated by many studies to be associated with cancer progression. Interestingly, only SP1 was also enriched within the 52-gene common gene list derived from gene-level method (data not shown). We then extracted 19 miRNAs with their predicted targets enriched within the differential gene lists from at least 4 of the 6 total datasets (see Additional file 14). Among these miRNAs, there are quite a few that have been previously reported to be aberrantly expressed in cancer or related to carcinogenesis, and have been proposed to function as a novel class of oncogenes or tumor suppressor genes [36-38]. Interestingly, some of them were also enriched within the 52-gene common gene list derived from gene-level method, although many of them were missed from the enrichment result of 52-gene common gene list (data not shown). Even for KEGG pathways [4], we extracted 6 KEGG pathways enriched within the differential gene lists from at least 3 out of the total 6 datasets (see Additional file 15). Many of these pathways such as integrin-mediated cell adhesion [39], MAPK signaling pathway [40], TGF-beta signaling pathway [41], and Wnt signaling pathway [42] have been implicated in tumor progression and carcinogenesis. However, none of these significant KEGG pathways were enriched within the 52-gene common gene list derived from gene-level method (data not shown).

These observations suggested that PPEP can not only identify additional terms or wider biological themes that may be missed by conventional gene-level based methods, but also may provide a way to cross-validate the results derived from the gene-level based methods since it should also include any such terms.

### Seeking unique biological themes specific for tissue types

PPEP can not only help identify common biological themes among different datasets or studies as described in the above case study, but also can help uncover uniqueness of a subset that is distinct from the rest of a dataset or study. Here, we describe a case study that looks for tissue-specific biological processes from the well known public microarray dataset derived from 79 human tissues from the Genomic Institute of the Novartis Research Foundation (GNF) described previously [43].

We first preprocessed the data by z-score transformation of the data and then sorted the genes into individual gene lists as highly expressed genes for each corresponding sample with the criteria that the z-score was no less than 1. Finally, we subjected these gene lists to the PPEP. The goal of this analysis is to look for those biological processes that are uniquely but consistently enriched in highly expressed genes within certain types of tissues. We chose to examine those biological processes that are shared amongst in the testis-related tissues but distinct from the rest of the tissues in the dataset. We chose the subset, since there are only 5 testis-related tissues (testis germ cells, testis interstitial, testis leydig cell, testis seminiferous tubule, and testis) in the dataset and testis-related biological processes are relatively unique and specific compared to other tissues. The analysis result should be easily interpreted based on their biological expectations.

We set up the pathway pattern extraction template exactly as illustrated in Figure 3 for annotations of GO biological processes, which allowed us to find the GO terms that are enriched in at least 4 of the 5 testis-related tissues but not enriched in at least 72 of 74 other tissues. We used slightly relaxed criteria for pathway pattern extraction in this case to avoid the possible situation where one sample may have some experimental noise, such as contamination, causing some relevant terms to be missed in our extracted patterned term lists as a result of the errors in one (in Inclusion samples: testis-related tissues) or two (in Exclusion samples or other tissues) samples. Another reason for the relaxed criteria is the fact that these 5 related tissues are not exactly the same tissues. Thus, our relaxed criteria may help overcome the diversity among these testis-related tissues but differentiate them as a group from other types of tissues. The pathway pattern extraction generated a list of 16 terms, which were visualized in a heatmap using the enrichment scores of each term for each sample (Figure 4, see Additional file 16 for the real data). Many of these 16 GO terms appeared to be quite specific to testis-related functions such as development of primary male sexual characteristics, fusion of sperm to egg plasma membrane, male gamete generation, male sex differentiation, spermatogenesis, which obviously are exactly what were expected from the analysis. Some non-testis tissue samples also had relatively high enrichment scores, possibly due to multiple or redundant functions of some genes that may work in other tissues or resulting from possible accidental enrichment due to the relatively relaxed selection criteria for genes (e.g., z-score approach) (Figure 4). Furthermore, most of these samples were only enriched in one or two terms except for Ciliary_Ganglion, which are enriched in 5 testis-related functional terms (see Additional file 16). However, as described in a separate manuscript, our newly developed method SLEPR may help rank these terms to position the most relevant terms at the top of the ranked list [22]. We feel that combining SLEPR and PPEP within the same application gives biologists access to a much richer breadth of information to use for exploratory hypothesis testing.

To compare the gene-level analysis with the PPEP method, we also obtained the common genes from the highly expressed gene lists of the 5 testis-related tissues and subjected them to enrichment analysis on GOBP terms. We retrieved the top 16 enriched GO terms and compared them with those 16 GO terms that were identified using PPEP. Interestingly, many of these top ranked 16 terms derived from common gene list-based approach in fact were also widely enriched in many non-testis tissues (data not shown), which is in contrast to the behavior of the 16 terms derived from the PPEP. This observation indicated that PPEP method showed a much more specific enrichment pattern in testis-related tissues than the common gene list-based approach. Similar to the case study with prostate cancer dataset described earlier, this observation indicates that the gene-level method appeared to have more difficulty to uncover the unique biological processes specific for testis-related tissues at the pathway-level compared to the PPEP method.

To explore what genes are associated with the extracted testis-specific GO terms, we used the interface shown in Figure 5 to retrieve the genes for some selected terms in a format of pairwise relations with the selected GO terms (see Additional file 2). We can then display the gene-term relationships in the context of GTANs shown in Figure 6. Although many associated genes are consistently expressed in all of the 5 testis-related tissues at a relatively higher level, there are other genes that are expressed only in some testis-related tissues (Figure 6).

We also used our PPEP to seek common and unique biological themes in two-class comparison in another case study (See Additional file 17 (Additional file 18 and 19)), which also showed great flexibility and capacity of the PPEP in such circumstances.

## Discussion and Conclusion

Many software tools begin with a gene list, either as differential genes, or genes with certain biological features or defined properties, and subject the list to enrichment analyses (typically overrepresentation analysis (ORA) [12,13] or GSEA [15,16]). Such analysis usually focuses on uncovering embedded biological implications in one gene list or a single data set.

Our high-throughput PPEP is designed to extend this analysis scheme into a comparative analysis scheme at the pathway-level for multiple gene lists or multiple HTP datasets. It is based on the assertion that it has the potential to integrate data from different, but related studies or even from different platforms. The advantage of the method was illustrated by the case study that used 6 prostate cancer datasets from the Oncomine database described in the result section. Recently, a meta-analysis method has made great progress trying to integrate data from different platforms by means of a summary statistics-based method in combination with FDR analysis and cross-validation [26,44,45]. However, it is still a gene-level based approach that primarily looks at the consistency of gene behavior across datasets. In contrast, our high-throughput PPEP is designed to compare genes of defined gene lists at the pathway-level to determine the enrichment levels of defined pathways or gene sets. The idea is to integrate data at the pathway-level for multiple gene lists or multiple HTP datasets without regard to the types of the platforms, technologies, and statistical methods that were applied to generate these datasets or gene lists. Thus, our results indicate that common or unique biological themes can effectively be captured at the pathway-level by means of conducting pattern analysis of the enrichment levels of functional terms, pathways, or gene sets across the datasets or gene lists. The genes associated with these patterned pathways or terms can then be retrieved, and the data behavior, as well as relationships with the terms, can be analyzed in the context of GTANs within the WPS environment [4]. The WPS environment allows for visualization of data behavior of multiple gene lists together [4].

As shown in the first case study in the Results section, our analysis scheme with PPEP effectively uncovers many common pathway-level signatures from the 6 prostate cancer datasets. From the analysis done within each individual gene list or dataset, some of these common pathway-level signatures may not be seen as significant, since they were not ranked at the very top in the enrichment results of each individual gene list of these independent studies. However, the consistencies and patterns of pathway-level enrichment that PPEP relies on adds weight to these terms and promotes their significance so that they become significant underlying biological themes across the independent studies. In contrast, using the common differential genes of these independent studies based on gene-level consistency as the sole-source for enrichment analysis, the derived pathway-level signatures may not be able to get significant enrichment scores. This observation illustrates the advantage of PPEP compared to conventional gene-level based enrichment methods in rationale. Our other case studies described in the Results section further substantiated the increased ability of PPEP to identify meaningful biological terms associated with input sets of gene lists derived from different sources, or with different technologies.

In comparison with many other gene-set based software tools, our PPEP has many unique features not found in a single application (Table 1). Some of the features such as pathway pattern extraction are only found in our pipeline as far as we have been able to determine. In a typical ORA method [46] and FCS method [47], the ranking of the "significant" over-represented terms can be misleading due to the fact that the real biologically relevant terms may not be necessarily ranked at the very top. This results from the fact that the ranking is purely based on the p-values or enrichment scores, which in some cases may largely be influenced by the numbers of genes annotated in the terms or the size of the gene list. Our pathway-level comparative analysis method looks at the patterns of enrichment status (whether enriched or not) that persistently exist across the gene lists. By design, it ignores the enrichment magnitude (or enrichment levels), which is more susceptible to the changes in the numbers of genes annotated in the terms or the size of the gene list, as long as the terms are enriched at significant level (e.g., p < 0.05 and ListHits >= 2). In other words, our method pays more attention to the terms that are consistently enriched across gene lists compared to which terms are mostly enriched in individual lists usually reported by individual ORA analysis (see Additional file 1 for more technical details of PPEP).

**Table 1:** Comparison of major features of PPEP in WPS with other related tools

	WPS PPEP	Babelomics	High-throughput GOminer	EASE/DAVID	GSEA
Number of gene lists to be analyzed at the same time for comparison	Multiple lists	1–2 lists	Multiple lists	one list a time	NA

Batch computation for multiple gene lists	Yes	No	Yes	No	No

Merging Batch Results for a matrix of data	Yes (*ES, ListHits, FDRs)	No	Yes (FDRs)	No	No

Annotation scopes	Many: Pathways; GO; miRNA Targets; TF targets; GSEA annotations	GO terms	Go terms	Many: Pathways, GO terms Interaction	GSEA annotations

Pattern extraction at pathway enrichment level	Yes	No	No	No	No

Provide matrix of data for clustering analysis at pathway level	*ES, ListHits, or FDRs	No	Only FDRs	No	No

Retrieve associated genes from selected terms	Yes	No	No	No	No

Network and data visualization of the associated genes and terms (GTANs)	Yes	No	No	No	No

Gene list sorting utility	Yes	No	No	No	No

Data manipulation utility	Yes	No	No	No	No

SLEPR pathway ranking method [22]	Yes	No	No	No	No

Group testing methods using gene sets	ORA	ORA	ORA	ORA	FCS

The Pathway Pattern Extraction Pipeline (PPEP) in WPS has many unique features not found in a single similar application. Five related tools (first row of the table) were selected for comparison of major features (first column of the table) with PPEP in WPS. The other 4 tools for comparison include: Babelomics (Fatigo) [13]; HTP GoMiner [14]; EASE/DAVID [12,46]; GSEA [15,16].

ORA: Over-representation analysis [21]; FCS: functional class scoring [47]; *ES: Enrichment score.

We also have evidence that our pathway pattern extraction method is more effective than the PTM-based (Pavlidis template matching) clustering method (TM4) [23] in uncovering patterned pathways or terms when measured by completeness and intuitiveness of resulting terms. Our pathway pattern extraction method can easily extract the complete set of patterned terms, whereas the PTM clustering method may have to test different p-values before reaching the best match (see Additional file 20). The main distinction is that the PTM clustering method is based on the actual values (how closely the values (i.e. ESs or enrichment scores) are related to each other) and our pathway pattern extraction is based on the categorized calls (enriched or not). In other words, our pathway pattern extraction looks for the patterned enrichment status (enriched or not) rather than the enrichment magnitude (how significant it is enriched in terms of the p-values or enrichment scores). Thus, our method may be more effective in picking up the patterns and more realistic in considering the enrichment status rather than the enrichment scores that may be influenced by many factors.

As demonstrated by our case studies using our PPEP, many commonly shared biological themes can be easily captured from multiple gene lists derived from related, but different studies. The same themes are not obvious when simply looking at the common genes shared by these lists at both the gene and pathway-level. In addition, the unique biological themes, which are specific to one or more gene lists, but not to others, can be uncovered as list-specific biological processes at the pathway-level. For example, for closely related tissues such as the 5 testis-related tissues (testis germ cells, testis interstitial, testis leydig cell, testis seminiferous tubule, and testis) in the case study using the GNF dataset [43], since they are, in fact, different tissues, it may be more appropriate to expect higher similarity in biological processes at pathway-level than at gene-level. Thus, the difference between testis-related tissues would be less profound at the pathway-level than that at gene-level. This notion has been extended to a novel approach that can be also applied to common class comparison analysis in our newly developed SLEPR method described in a separate manuscript [22], which considers sample-level variations of individual genes.

Many efforts have been made to look for biological themes using networks of associated genes. These are largely based on curated gene-gene relationships from the literature [9,10]. The pure pathway/network approach often encounters issues of complexity of networks that are generated from a relatively large list of statistically significant genes. Without considering the major trends for biological themes embedded in the gene list, it would be rather hard to derive biological insights from these networks. In a more complicated situation where there are multiple gene lists from different studies and one wishes to compare the embedded biological themes from them, directly applying these gene lists into the networks would be rather difficult to sort out the underlying biological themes. This stems from a lack of an accepted and robust method for comparing the derived network of each list. Until such a method can be tested, it would be a better choice to use our pathway pattern extraction approach looking for common and unique biological themes first, before subjecting the associated genes of the selected terms to network analysis. This would not only simplify the size of the gene list used for the network analysis, but also may use more relevant genes to create a more meaningful network that may provide better insights into the underlying biological themes.

Before describing PPEP in this manuscript, it has been widely used for analysis of many of our collaborators' data including microarray datasets, mass spectrometry data, data from a genetic screening for functional impact study of virus insertion sites in the genome, lists of genes with specific features embedded in their sequences [[48-54], many unpublished studies]. It has successfully uncovered many underlying biological themes that gene-level methods might have missed or failed to reveal. The analysis results have been included in many published studies with our collaborators using different biological systems including breast cancer [48], prostate cancer [49,50], yeast genetics [51], malaria drug-resistance studies [52], colorectal cancer [53] and Parkinson disease [54] where it helped uncover underlying biological themes. Given the insights derived from the analysis using the PPEP, we believe that the new analysis scheme provided by this pipeline will be very helpful in compensating for some of the limitations of gene-level based analysis, and provide a valuable perspective of data sets from a pathway point of view. With a better coverage of associated genes for desired pathways and biological processes, our method yields a better chance to envision a more complete picture of biological themes (e.g., behavior of genes at the pathway-level or as a functional group for each individual samples) despite individual variations in gene lists or from datasets derived from independent studies with different rationales, technology platforms, and/or sampling populations.

## Availability and requirements

**Project name**: Pathway pattern extraction pipeline (PPEP) in WPS

**Project home page**: WPS with PPEP and corresponding databases (WPS version 2) can be downloaded from WPS homepage [55]. The demo movies for PPEP can be accessed through the demo page of the WPS homepage.

**Operating system**: Microsoft Window 2000 or XP

**Programming language**: Microsoft Visual Basic 6

**Other requirements**: Built on the platform of WPS [55].

**License**: Free to academics; distributed through license agreement

**Any restrictions to use by non-academics**: commercial license needed

## List of abbreviations used

WPS: WholePathwayScope; ES: Enrichment Score; GTAN: Gene-Term Association Network; HTTP: High Throughput; KEGG: Kyoto Encyclopedia of Genes and Genomes; GO: Gene Ontology; GOBP: Gene Ontology: Biological Processes category; PPEP: Pathway Pattern Extraction Pipeline; ORA: Over-representation analysis; FCS: functional class scoring; GSEA: Gene Set Enrichment Analysis.

## Authors' contributions

MY carried out the design and implementation of the PPEP in initially developed WPS [4] and drafted the manuscript. UM participated in collect and assembly of WPS database. AC participated in collect and assembly of WPS database. RMS helped the design and funding for PPEP in ABCC, and drafted the manuscript. All authors read and approved the final manuscript.

## Supplementary Material

Additional file 1 **More details of PPEP**. More technical details, features, and implementations of PPEP. Click here for file

Additional file 2 **The gene-term pairwise relation table**. The gene-term pairwise relation table that was retrieved using an interface as illustrated in Figure 5. Such a file content can be used in WPS to create the GTAN (gene-term association network). Click here for file

Additional file 3 **The interface of data manipulation utilities**. The image file showing the interface of data manipulation utilities in WPS. Click here for file

Additional file 4 **The interface for data scoring and lists sorting**. The image file showing the interface for data scoring and lists sorting for SLEPR method [22] available in PPEP. Click here for file

Additional file 5 **Common differential genes among all datasets**. list of 52 genes that were obtained as common differential genes among all of the 6 datasets using gene pattern extraction feature in WPS in the prostate cancer case study. Click here for file

Additional file 6 **common GSEA annotation terms among all datasets**. list of 331 terms that were obtained as common GSEA annotation terms among all of the 6 datasets using pathway pattern extraction feature in PPEP of WPS in the prostate cancer case study. Click here for file

Additional file 7 **Enrichment result of GSEA annotations for the 52 common differential genes**. The full enrichment result of GSEA annotations for the 52 genes that were obtained as common differential genes among all of the 6 datasets in prostate cancer case study. Click here for file

Additional file 8 **Pathway-level enrichment patterns of commonly enriched GSEA terms**. The heatmap of pathway-level enrichment patterns of the 33 commonly enriched GSEA terms among the differential genes of 6 datasets in prostate cancer case study that are directly involved in cancer-related processes. Click here for file

Additional file 9 **Comparison of results of PPEP and gene-level method for cancer-related terms**. The merged table in between the 33 cancer-related terms enriched commonly in 6 datasets of prostate cancer case study and the terms that have at least one gene out of the 52-gene common gene list, which shows that only 7 of the 33 terms are enriched in 52-gene list. Click here for file

Additional file 10 **Terms that are enriched consistently in all datasets**. List of the 44 GO terms derived from PPEP that are enriched consistently in the differential genes of all 6 datasets in prostate cancer case study. Click here for file

Additional file 11 **Full enrichment result of GOBP annotations for the 52 common differential genes**. The full enrichment result of GOBP annotations for the 52 genes that were obtained as common differential genes among all of the 6 datasets in prostate cancer case study. Click here for file

Additional file 12 **Pathway-level enrichment patterns of the commonly enriched GOBP terms**. The heatmap of pathway-level enrichment patterns of the 44 commonly enriched GOBP terms among the differential genes of 6 datasets in prostate cancer case study. Many of these terms are apoptosis-related. Click here for file

Additional file 13 **Transcription factor target enrichment patterns among the differential genes**. The heatmap of transcription factor target enrichment patterns among the differential genes of 6 datasets in prostate cancer case study. The 6 gene lists are enriched consistently with predicted targets of the corresponding transcription factors. Click here for file

Additional file 14 **miRNA target enrichment patterns among the differential genes**. The heatmap of miRNA target enrichment patterns among the differential genes of 6 datasets in prostate cancer case study. The 6 gene lists are enriched consistently with predicted targets of the corresponding miRNAs. Click here for file

Additional file 15 **KEGG pathway enrichment patterns among the differential genes**. The heatmap of KEGG pathway enrichment patterns among the differential genes of 6 datasets in prostate cancer case study. At least 3 out of the 6 gene lists are enriched for the corresponding KEGG pathways in heatmap. Click here for file

Additional file 16 **Data details of heatmap in Figure **4. The data matrix for the heatmap shown in Figure 4. Click here for file

Additional file 17 **Details of a case study in two-class comparison**. detailed description of a case study of the PPEP to seek common and unique biological themes in two-class comparison. Click here for file

Additional file 18 **GOBP term enrichment patterns in class comparison of muscle vs. testis**. The heatmap of GOBP term enrichment patterns in class comparison case study. The 5 GOBP terms are commonly enriched in all 4 muscle-related tissues but not in any of the testis-related tissues. Click here for file

Additional file 19 **GOBP term enrichment patterns in class comparison of testis vs. muscle**. The heatmap of GOBP term enrichment patterns in class comparison case study. These GOBP terms are commonly enriched in all 5 testis-related tissues but not in any of the muscle-related tissues. Click here for file

Additional file 20 **advantage of PPEP method over the PTM method in TM4**. Slides showing the advantage of PPEP method compared to PTM clustering method in TM4 [23]. Click here for file
