# Supplementary material for: Seeking unique and common biological themes in multiple gene lists or datasets: pathway pattern extraction pipeline for pathway-level comparative analysis
Source: BMC Bioinformatics. 2009 Jun 29;10:200. doi: 10.1186/1471-2105-10-200 (PMC2709625; doi:10.1186/1471-2105-10-200)
Supplement: Additional file 1 — More details of PPEP. More technical details, features, and implementations of PPEP. [file 1471-2105-10-200-S1.doc]

***Batch compute Fisher’s exact test and merge result files into enrichment scores matrix***

As illustrated at the first step of the pipeline in Figure 1, PPEP has extended the enrichment analysis of the individual gene lists in the original WPS program as described previously [4] into a batch mode to compute for the enrichment statistics of multiple gene lists simultaneously and collate the results thereafter. As shown in the one of the PPEP interfaces in Figure 2, to do the batch-mode computation of Fisher’s exact test, users have options to select different functional categories such as GO Biological Processes [4], GSEA annotation [16], BioCarta pathways [4]. To enhance its capacity to deal with different statistics of enrichment, PPEP provides the options of calculating and merging the result files for their enrichment scores (ESs; derived from p-values of Fisher’s exact test by a formula of –log10(p-value)), ListHits (number of genes from the gene list are annotated for the corresponding term), or FDRs (false discovery rates as q-values estimated by permutation methods [22], merging in a similar way as p-values). Appropriate filtering can be applied as desired: typically, ListHits<2 or p-value>0.05 can be used to floor the ESs to 0, which has the effect of depreciating the terms with single gene hit from the list or with p-value beyond the conventional significant range. Otherwise, –log_10_(p-value) will be directly applied to each p-values to get the corresponding ESs.

***Pathway-level pattern extraction and pathway/term ranking for significance***

Once a matrix of enrichment scores (ESs, or ListHits, FDRs) is created, pattern analysis can be applied to it in a way similar to the regular data matrix. The popular heatmap-type of analysis, including all types of clustering analyses and PTM-based pattern extraction (TIGR), can be used to search for patterned pathways or terms from the ESs matrix. Due to the abundance of related clustering analysis tools [1, 23, 24], our analysis pipeline does not directly provide the clustering analysis functionality beyond creating the data matrix of pathway-level data such as a matrix of Enrichment Scores (ESs) of all the desired gene lists. Beyond the conventional clustering analysis methods, as shown in Figure 3, PPEP provides a pathway-level pattern extraction interface that allows users to easily set up desired template for pathway-level pattern extractions. Compared to the original gene-level pattern extraction feature available in the original version of WPS, this pathway-level pattern extraction interface tends to be more powerful and flexible in terms of the data that it deals with (e.g., gene-level, and pathway-level data), as well as the more comprehensive patterns it can handle and the options it offers (Figure 3: Inclusion and Exclusion classes with criteria and extract options, and options for SLEPR method [22]). Real examples were also provided in the Results section to illustrate the usage of these options.(Figure 3 and 4)

***Retrieve associated genes from selected terms***

Once desired terms are obtained from pathway pattern analysis, the associated genes for these terms from any of the original gene lists can be retrieved using an interface from the PPEP, as illustrated in Figure 5. The relationships of the retrieved associated genes and terms can be saved to files (see Additional file 2) and viewed graphically as Gene-Term Association Networks (GTANs) as described previously (Figure 6; [4]). Thus, patterned pathways or terms can be used to retrieve the involved genes from one or more gene lists and the data behavior of those genes can be easily studied in the context of GTANs for association relations with the patterned pathways or terms. Besides these core interfaces, PPEP also provides several data manipulation utilities to facilitate the data analysis (see Additional file 3 and 4).

***Rationale, Class Setting and Extraction Options in Pathway Pattern Extraction in PPEP***

Although Enrichment Scores (ESs) may be also impacted by the size of gene list and total number of genes, compared to ListHits, the ESs derived from the p-values of Fisher’s exact test. Therefore, ESs should be relatively more stable in terms of its capacity of reflecting the enrichment status (whether it is enriched or not for a pathway or gene set) rather than the actual values of ESs. In this sense, it seems to be more reasonable to compare the ESs among the multiple gene lists for each pathway or gene set in terms of enrichment status, since the suggested Fishers’ exact test is already contained in this metric. In other words, PPEP focused more on the enrichment status (enriched or not: with some reasonable and conventional cutoff such as p<=0.05 or ES>=1.3 and help of ListHits (ListHits >=2), we can effectively categorize the data) rather than the enrichment magnitude (the values of ESs). In fact, that’s exactly what PPEP relies on to perform the pathway-level pattern extraction and look for more of a pattern of enrichment status rather than the magnitude of the enrichment level. This is basically the exploratory feature of PPEP. That’s why we called it as Pathway-level Pattern Extraction as opposed to the clustering-based profiling, which would actually rely on the enrichment status as opposed to the values of ESs. As we mentioned in the discussion section of the manuscript, the analysis scheme offered by PPEP has been widely used and successfully provided biological insights from the data as published in many of our collaborators’ work (Reference 46-52).

On the other hand, in a more defined data analysis for evaluation of so-called pathway-level consistency for a dataset with typical two-class contrast, the ESs have been successfully used as basis to directly compare across individual samples. This provided insights from the datasets at pathway-level (see our published work on SLEPR method: Yi and Stephens 2008, PLoS ONE 2008, 3(9): e3288). That’s why we felt that the ESs can be appropriately compared across the gene lists or datasets in terms of enrichment status rather than enrichment magnitude.

PPEP is intended to be an exploratory tool in a comprehensive way that allows the users to compare multiple genes lists and datasets at pathway-level for enrichment levels (ESs), ListHits, FDRs of Enrichment, and eventually to evaluate pathway-level patterns across these gene lists in terms of underlying biological significance. The ability to compare the ListHits, ESs, FDRs at the same time would be a great advantage for data analysis in PPEP. This would allow the users to have a complete picture of data behavior (i.e., how many impacted genes in the pathway (ListHits), at what level the pathway was impacted (ESs for significance of enrichment status), how likely such impact would not be the random event compare to other pathways (FDRs)) for given conditions (Statistical criteria to derive the gene lists).

The data source file selected for Pathway Pattern Extraction can be a Microsoft Access file, a text file or a Microsoft Excel file. For a Microsoft Access or Excel file, one of the tables or spreadsheets can be selected further as the actual input for data source, respectively. Then the lists in columns of the data source matrix can be selected as for either the Inclusion class or the Exclusion class. As described previously [22], the Inclusion class is referred to as the class of gene lists that would meet the setting criteria, whereas the Exclusion class as a class of gene lists that would not meet the setting criteria. In other words, pathway-level pattern extraction in PPEP usually is intended to retrieve the terms that would meet the criteria of Inclusion class samples but not meet the criteria of Exclusion class samples. Although the individual Inclusion samples and Exclusion samples usually have the same criteria, they can have their own criteria that can be different from other individual samples. The pathway-level pattern extraction is done based on these Inclusion and/or Exclusion criteria. For example, for strict pattern extraction (without Extraction options for both Inclusion samples and Exclusion samples, see below), a term or pathway will be selected as patterned terms or pathways if the enrichment scores in inclusion samples for this term meet exactly with their corresponding Inclusion criteria and the enrichment scores in Exclusion samples for this term do not meet the exclusion criteria. The interface also provides Extraction options for both Inclusion samples and Exclusion samples so that the terms with a portion of all Inclusion samples or Exclusion samples matching the criteria can be extracted as the patterned terms.

***Extended database contents***

In the first version of WPS internal database [4], we only covered Gene Ontology (GO), Biocarta pathways, KEGG pathways. Now in the new version, we extended the database contents to cover many new categories: GSEA annotations from MSigDB (Human, Mouse)[16], protein-protein interaction (Human, Mouse, Yeast) (comprehensively curated from multiple protein-protein interaction databases, e.g., BIND, DIP etc., and kindly provided by Dr. Richard Lempicki’s group), predicted miRNA targets (Human, Mouse, curated from www.targetscan.org), in-house predicted transcription factor targets using consensus bind sites (Human, Mouse), pfam protein families (Human, Mouse), PIR protein families (Human, Mouse), Genetic Association database (Human, Mouse) [4], partial diseas

***Data manipulation features and utility interfaces***

To help the analysis using PPEP, several data manipulation utilities (see Additional file 3) were available for users to transform data (e.g., Z-score transformation, log transformation). After data transformation, genes can be sorted from a data matrix into lists using some criteria such as Z-score-based criteria, which was shown as an example for GNF dataset [26] in result section. There is also an interface specifically designed for scoring and sorting the genes into sample-level differentiated genes using MADe-based cutoff criteria in SLEPR method, as described in a separate manuscript [22] (see Additional file 4)

The pipeline can not only handle all these file types in any steps of the analysis scheme, but also allow easy insertion of data derived from other applications as well. This interface can be also applied to a newly developed method called SLEPR [22], which was implemented as a part of the analysis pipeline.
