# Supplementary figures and images for: Seeking unique and common biological themes in multiple gene lists or datasets: pathway pattern extraction pipeline for pathway-level comparative analysis
Source: BMC Bioinformatics. 2009 Jun 29;10:200. doi: 10.1186/1471-2105-10-200 (PMC2709625; doi:10.1186/1471-2105-10-200)

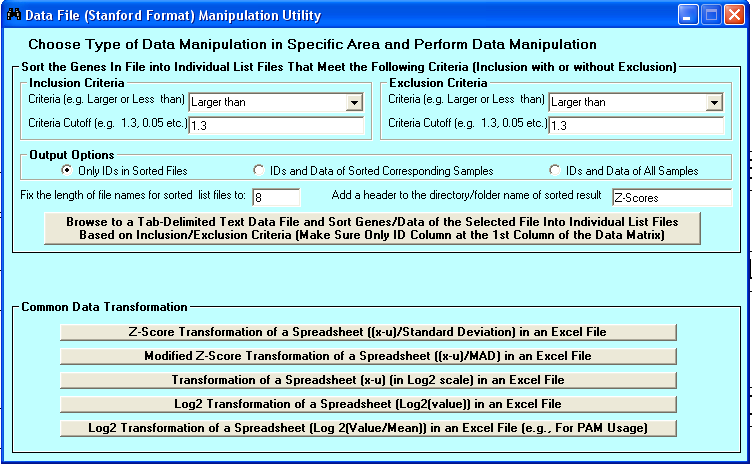

Supplement: Additional file 3 — The interface of data manipulation utilities. The image file showing the interface of data manipulation utilities in WPS. [file 1471-2105-10-200-S3.tiff]

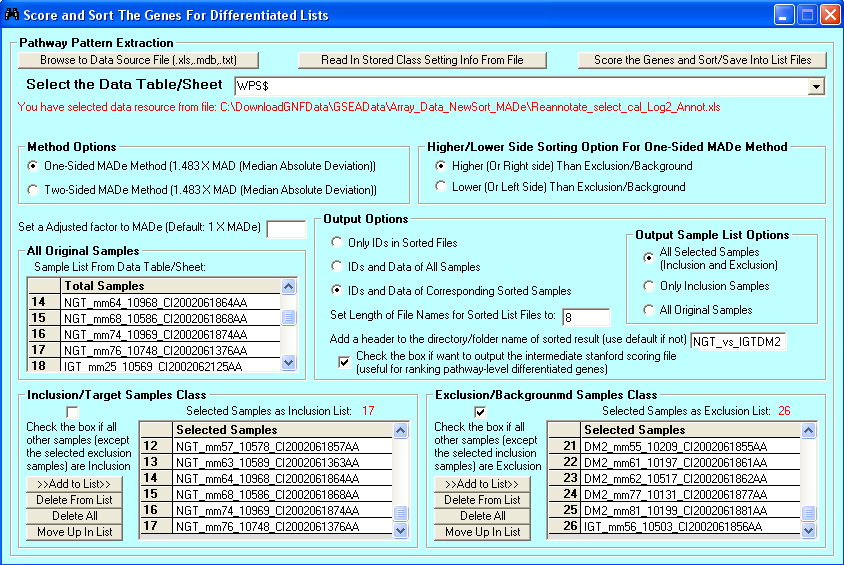

Supplement: Additional file 4 — The interface for data scoring and lists sorting. The image file showing the interface for data scoring and lists sorting for SLEPR method [22] available in PPEP. [file 1471-2105-10-200-S4.tiff]

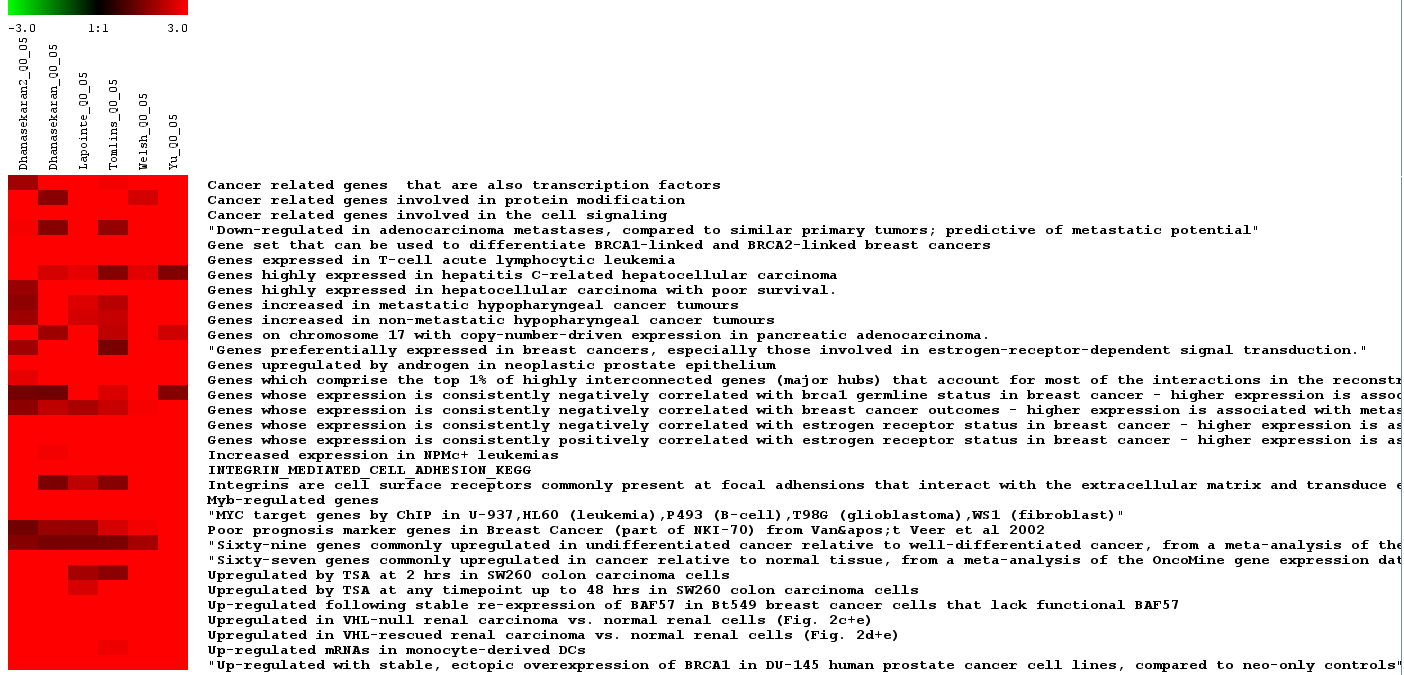

Supplement: Additional file 8 — Pathway-level enrichment patterns of commonly enriched GSEA terms. The heatmap of pathway-level enrichment patterns of the 33 commonly enriched GSEA terms among the differential genes of 6 datasets in prostate cancer case study that are directly involved in cancer-related processes. [file 1471-2105-10-200-S8.tiff]

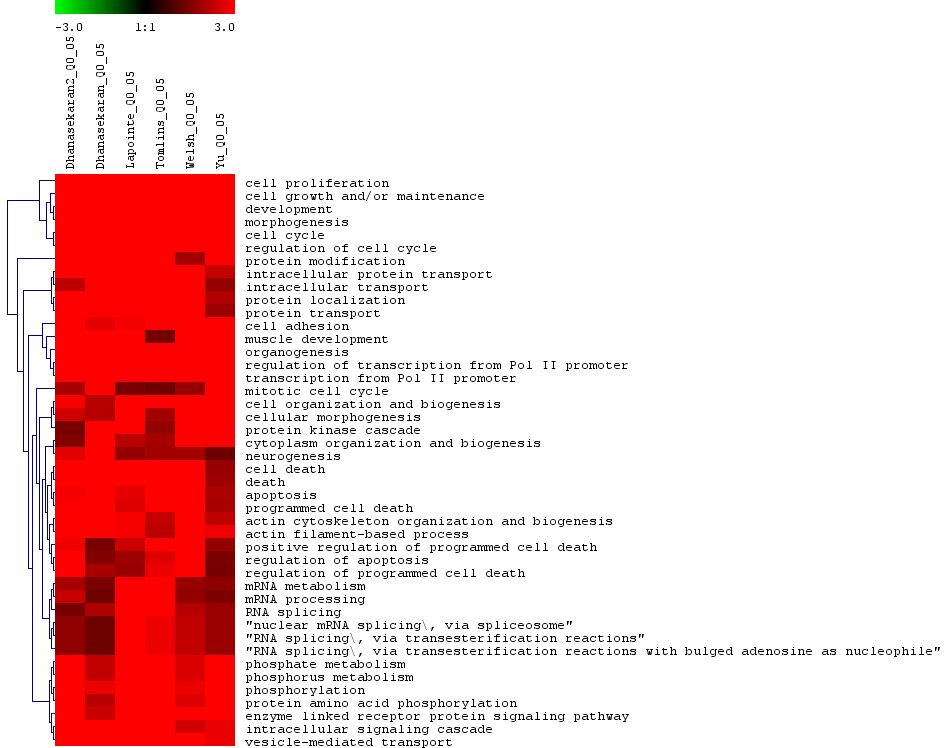

Supplement: Additional file 12 — Pathway-level enrichment patterns of the commonly enriched GOBP terms. The heatmap of pathway-level enrichment patterns of the 44 commonly enriched GOBP terms among the differential genes of 6 datasets in prostate cancer case study. Many of these terms are apoptosis-related. [file 1471-2105-10-200-S12.tiff]

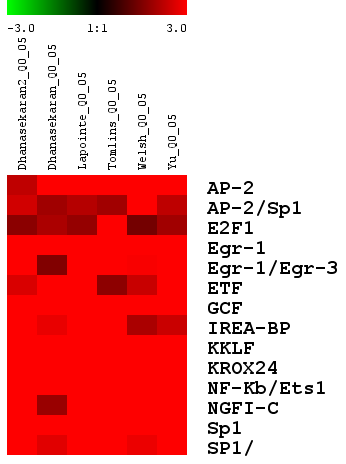

Supplement: Additional file 13 — Transcription factor target enrichment patterns among the differential genes. The heatmap of transcription factor target enrichment patterns among the differential genes of 6 datasets in prostate cancer case study. The 6 gene lists are enriched consistently with predicted targets of the corresponding transcription factors. [file 1471-2105-10-200-S13.tiff]

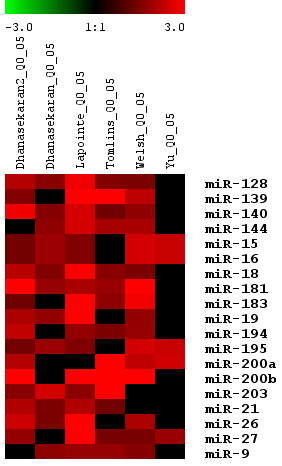

Supplement: Additional file 14 — miRNA target enrichment patterns among the differential genes. The heatmap of miRNA target enrichment patterns among the differential genes of 6 datasets in prostate cancer case study. The 6 gene lists are enriched consistently with predicted targets of the corresponding miRNAs. [file 1471-2105-10-200-S14.tiff]

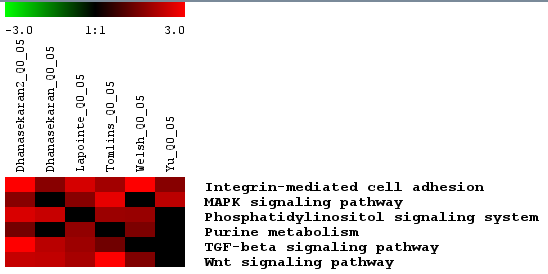

Supplement: Additional file 15 — KEGG pathway enrichment patterns among the differential genes. The heatmap of KEGG pathway enrichment patterns among the differential genes of 6 datasets in prostate cancer case study. At least 3 out of the 6 gene lists are enriched for the corresponding KEGG pathways in heatmap. [file 1471-2105-10-200-S15.tiff]

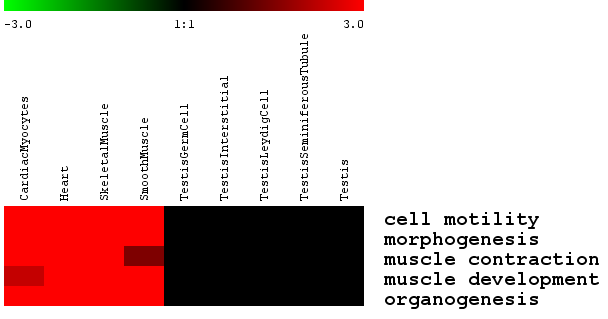

Supplement: Additional file 18 — GOBP term enrichment patterns in class comparison of muscle vs. testis. The heatmap of GOBP term enrichment patterns in class comparison case study. The 5 GOBP terms are commonly enriched in all 4 muscle-related tissues but not in any of the testis-related tissues. [file 1471-2105-10-200-S18.tiff]

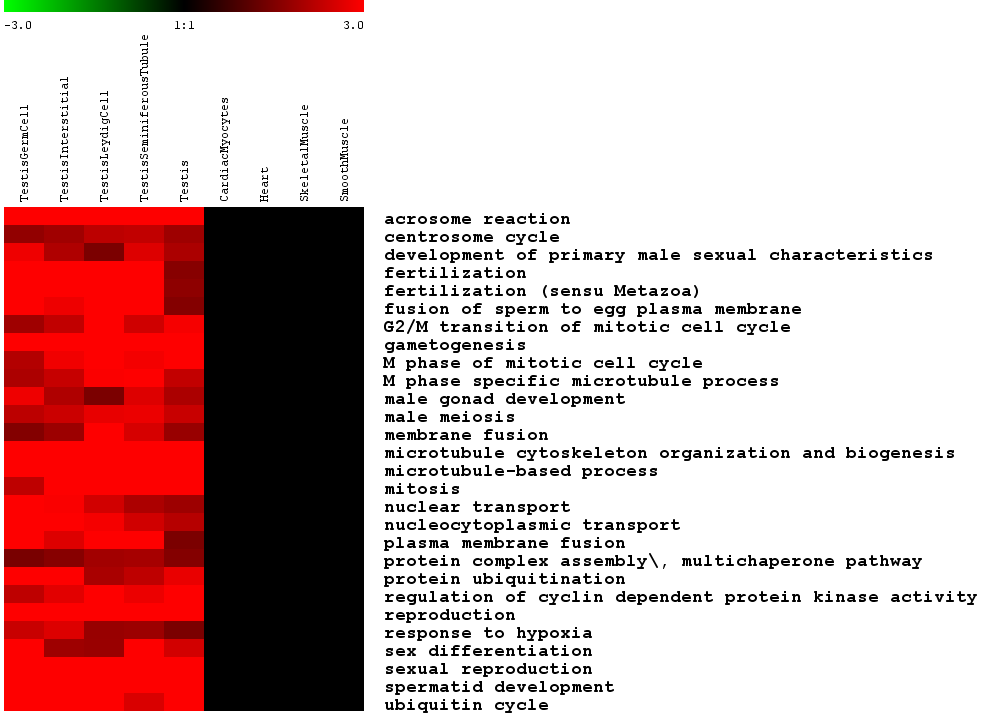

Supplement: Additional file 19 — GOBP term enrichment patterns in class comparison of testis vs. muscle. The heatmap of GOBP term enrichment patterns in class comparison case study. These GOBP terms are commonly enriched in all 5 testis-related tissues but not in any of the muscle-related tissues. [file 1471-2105-10-200-S19.tiff]
