# Supplementary material for: Seeking unique and common biological themes in multiple gene lists or datasets: pathway pattern extraction pipeline for pathway-level comparative analysis
Source: BMC Bioinformatics. 2009 Jun 29;10:200. doi: 10.1186/1471-2105-10-200 (PMC2709625; doi:10.1186/1471-2105-10-200)
Supplement: Additional file 17 — Details of a case study in two-class comparison. detailed description of a case study of the PPEP to seek common and unique biological themes in two-class comparison. [file 1471-2105-10-200-S17.doc]

***Case study: Seeking common and unique biological themes in class comparison***

In microarray studies, it is very common to use a two-class comparison (e.g., Tumor versus normal, different tissue comparison) and look for difference between the two classes. This is usually done at the gene level by looking for the differential genes as a representation of the difference between the two classes. However, given the possibility of diverse expression of many genes even in samples of the same class due to genetic variation, it seems likely that there would be additional insights gained by performing this analysis at the pathway level with PPEP. To address this question, we took only a subset of the GNF microarray tissue dataset described in the previous section including only the 5 testis related tissues and 4 muscle-related tissues (cardiac myocytes, heart, skeletal muscle, and smooth muscle). We globally applied z-score transformation to the 9-sample dataset, and then sorted the genes from the transformed data matrix into individual gene lists as relatively highly expressed genes for each corresponding samples with criteria of z-score no less than 1, similarly using the data manipulation interface (see Additional file 3). We then applied the PPEP to these gene lists (highly expressed in corresponding tissue samples of the 5 testis-related tissues and the 4 muscle-related tissues). In order to get unique GO terms of biological processes for each type of tissues, we did pathway pattern extraction using the interface shown in Figure 3. Under stringent extraction criteria, we found 5 shared GO terms that are commonly enriched in all 4 muscle-related tissues, but not enriched in any of the testis-related tissues (see Additional file 18). Two of them: muscle contraction and muscle development are obviously specific for muscle-related tissues. Although the other three are relatively generic, they are still quite relevant to muscle’s functions. Similarly, we found many GO terms that are unique but are consistently enriched in the gene lists of testis-related tissues as testis-related processes (see Additional file 19).

When we looked for common GO terms between these two groups of tissues, only one GO term of biological processes (development) is enriched in all gene lists of the 9 samples. With use of the Extraction option, we found that there are 7 common terms enriched in 7 out of the total 9 samples including cell cycle, cell proliferation, and transcription from Pol II promoter (data not shown).
