# Supplementary material for: Seeking unique and common biological themes in multiple gene lists or datasets: pathway pattern extraction pipeline for pathway-level comparative analysis
Source: BMC Bioinformatics. 2009 Jun 29;10:200. doi: 10.1186/1471-2105-10-200 (PMC2709625; doi:10.1186/1471-2105-10-200)
Supplement: Additional file 20 — advantage of PPEP method over the PTM method in TM4. Slides showing the advantage of PPEP method compared to PTM clustering method in TM4 [23]. [file 1471-2105-10-200-S20.ppt]

## Slide 1
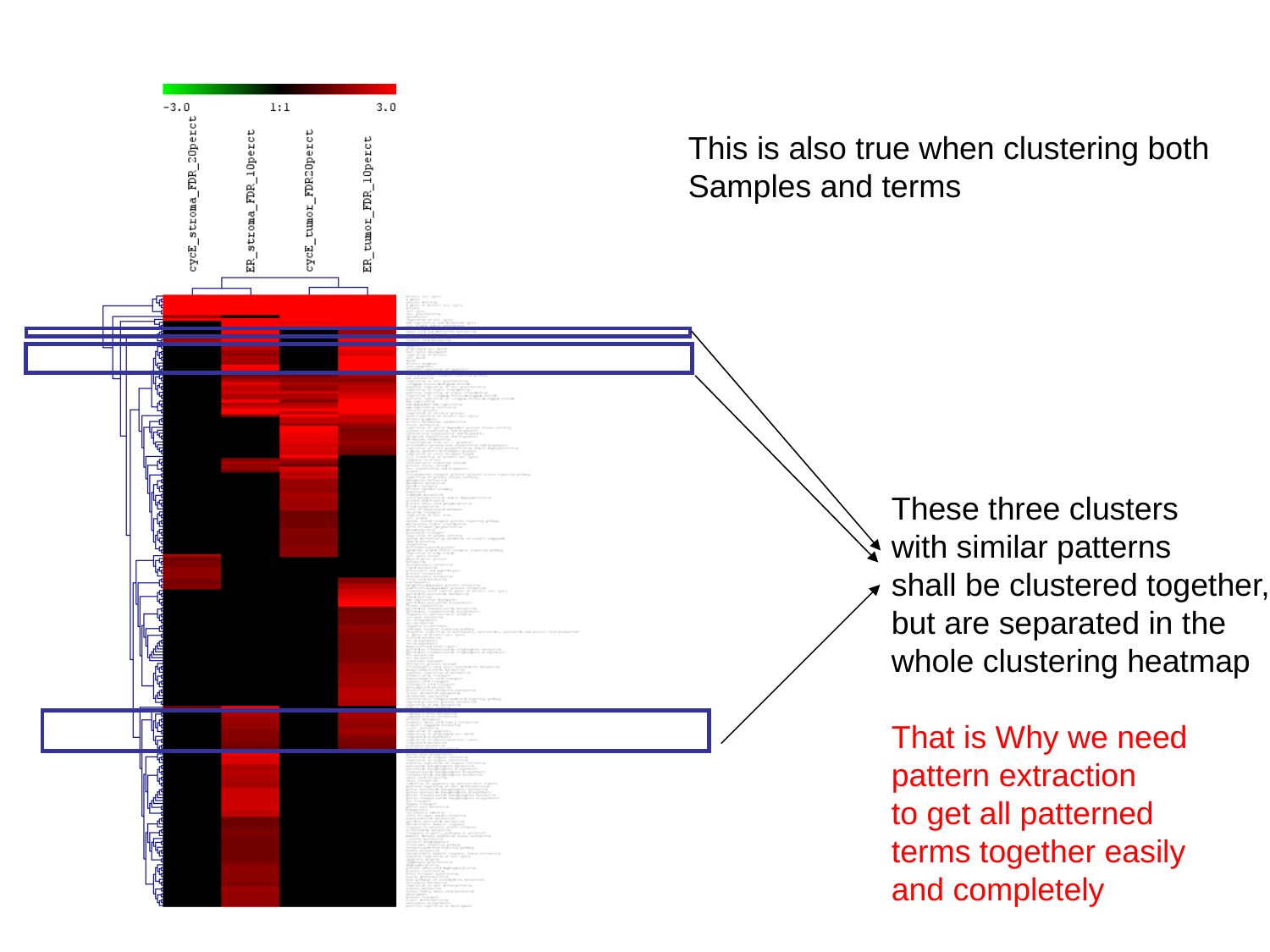

This is also true when clustering both
Samples and terms
These three clusters
with similar patterns
shall be clustered together,
but are separated in the
whole clustering heatmap
That is Why we need
pattern extraction
to get all patterned
terms together easily
and completely

## Slide 2
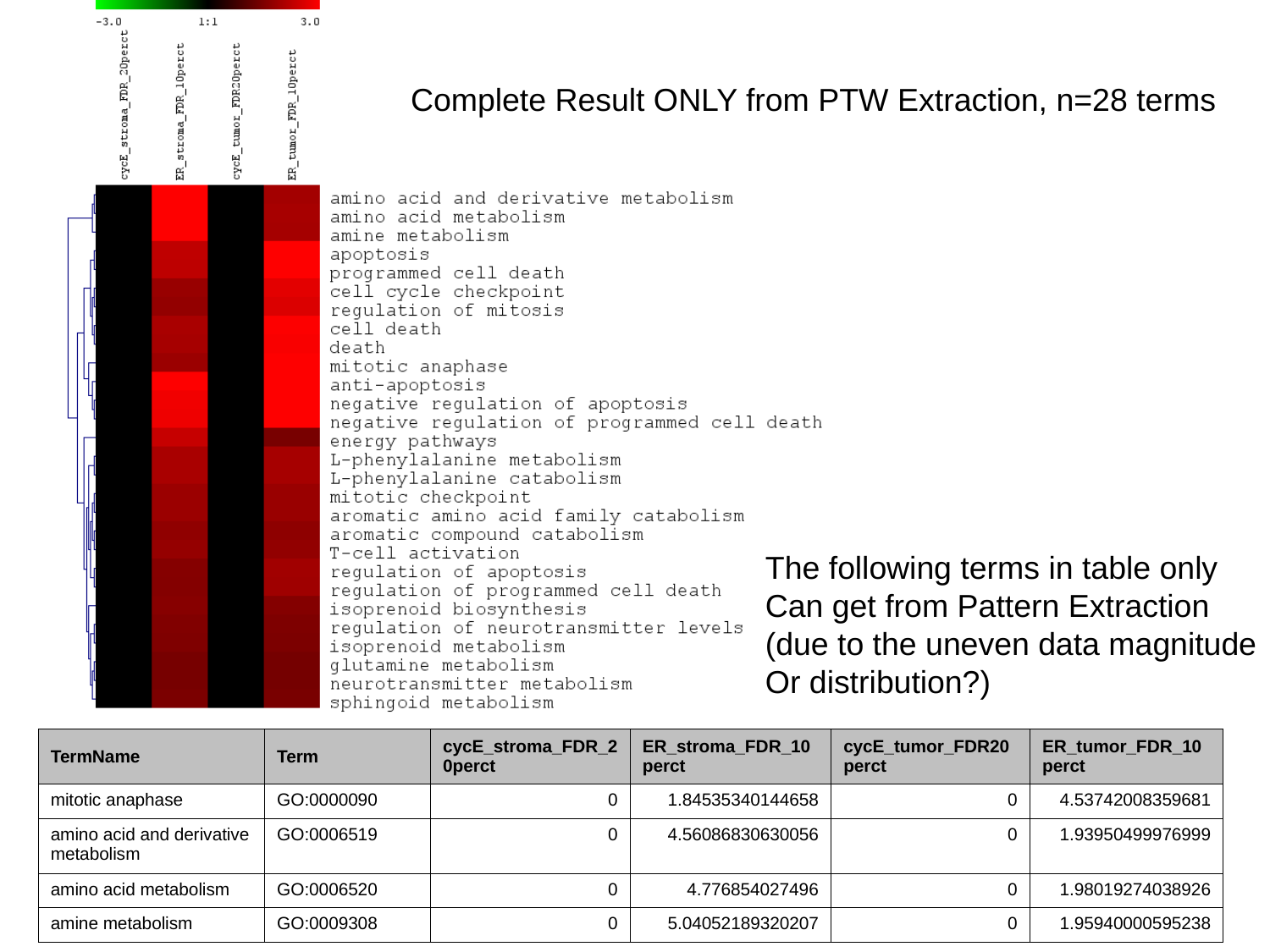

Complete Result ONLY from PTW Extraction, n=28 terms
The following terms in table only
Can get from Pattern Extraction
(due to the uneven data magnitude
Or distribution?)
| TermName | Term | cycE\_stroma\_FDR\_20perct | ER\_stroma\_FDR\_10perct | cycE\_tumor\_FDR20perct | ER\_tumor\_FDR\_10perct |
| --- | --- | --- | --- | --- | --- |
| mitotic anaphase | GO:0000090 | 0 | 1.84535340144658 | 0 | 4.53742008359681 |
| amino acid and derivative metabolism | GO:0006519 | 0 | 4.56086830630056 | 0 | 1.93950499976999 |
| amino acid metabolism | GO:0006520 | 0 | 4.776854027496 | 0 | 1.98019274038926 |
| amine metabolism | GO:0009308 | 0 | 5.04052189320207 | 0 | 1.95940000595238 |
